# Purpura Fulminans Following Toxic Epidermal Necrolysis-Like Presentation of Spontaneous Linear IgA Bullous Disease

**DOI:** 10.7759/cureus.12989

**Published:** 2021-01-29

**Authors:** Michael Tassavor, Bryan Tassavor, Ameen Al Awadhi

**Affiliations:** 1 Dermatology, Mount Sinai Hospital, New York, USA; 2 Dermatology, Boston University School of Medicine, Boston, USA

**Keywords:** linear, iga, purpura fulminans, necrosis, dic, toxic epidermal necrolysis, ten

## Abstract

Purpura fulminans (PF) is a life-threatening disease of cutaneous microvascular thrombosis and hemorrhagic necrosis. Linear IgA bullous disease (LABD) is an autoimmune disease of subepidermal blistering. We present the first known case of PF following a toxic epidermal necrolysis-like presentation of spontaneous LABD in a 70-year-old female.

## Introduction

Purpura fulminans (PF) is characterized by cutaneous microvascular thrombosis and hemorrhagic necrosis secondary to quantitative or functional deficiency of proteins C, S, and antithrombin III. In acute infectious PF, the most common type, infection attenuates thrombomodulin by cleaving it from the endothelial surface. This impairs protein C activation and leads to disseminated intravascular coagulation (DIC) [1,2].

Linear IgA bullous disease (LABD) is characterized by subepidermal blisters and linear deposition of immunoglobulin A (IgA) along the basement membrane zone (BMZ) on direct immunofluorescence (DIF). It is divided into a spontaneous and drug-induced form, the latter of which is most frequently seen with vancomycin. Though usually associated with polycyclic lesions in children, LABD has a much more variable presentation in adults, even mimicking toxic epidermal necrolysis (TEN) [3,4].

PF has been noted following TEN [5]. We present the first known case of PF following a TEN-like presentation of LABD.

## Case presentation

A 70-year-old female was brought to the Emergency Department (ED) with altered mental status after an unwitnessed fall, of which she had no recollection. The patient complained of bilateral leg pain from blistering lesions on her lower extremities. She denied any recent history of new drugs in the past year, and had long been taking apixaban, furosemide, and valsartan. No other collateral information was available. She was intubated for airway protection after workup revealed cardiogenic and septic shock. She was given vancomycin and aztreonam and admitted to the cardiac care unit along with a dermatology consult.

**Figure 1:**
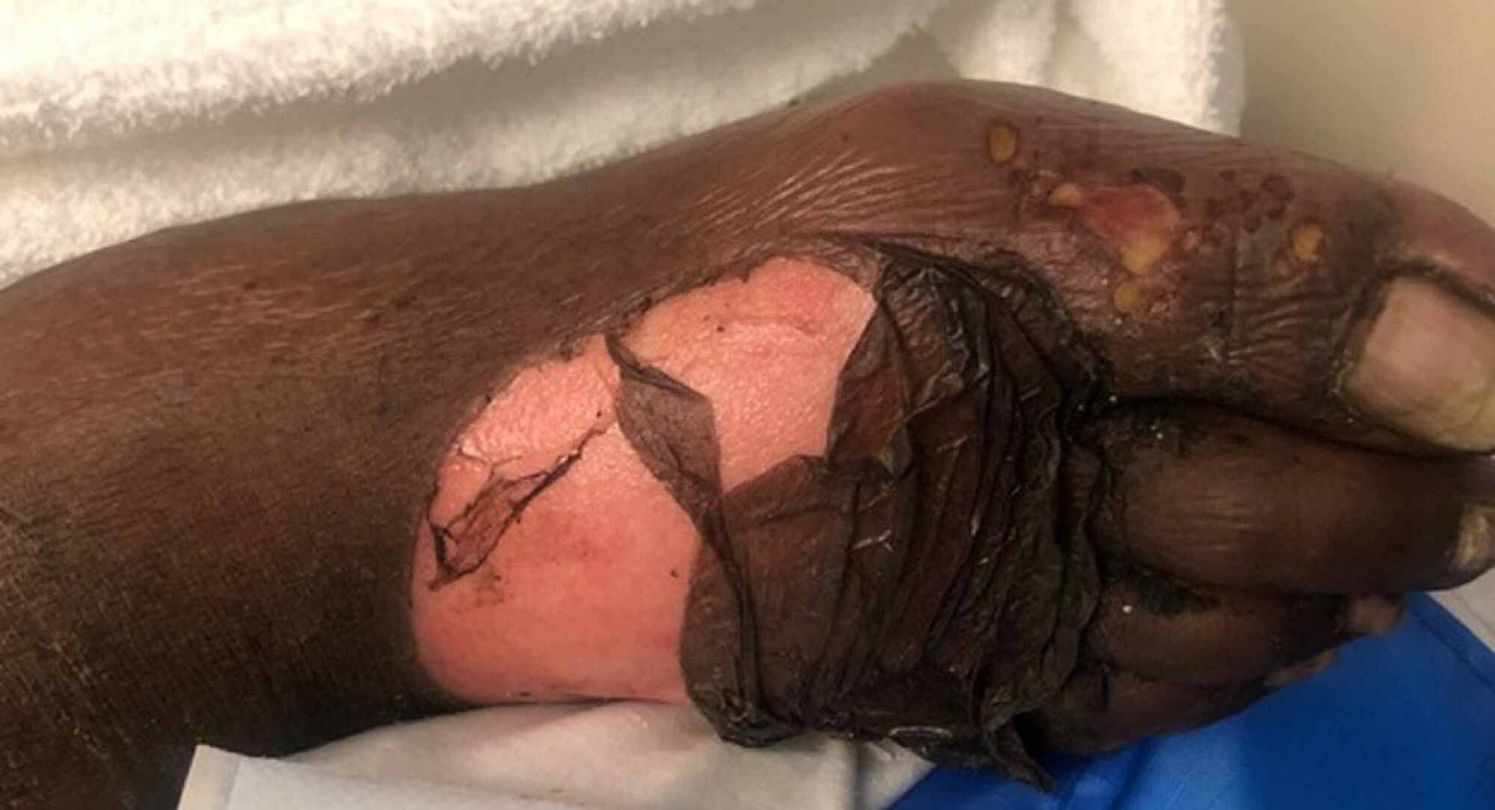
Right foot on day one.

On examination, she was found to have several areas of full thickness epidermolysis limited to lower extremities without mucocutaneous involvement or Nikolsky’s sign (Figure 1). Two punch biopsies of a bullae on her left leg were sent for hematoxylin and eosin staining and DIF. Her blood cultures from the ED revealed gram negative sepsis by the next day, with heavy growth of *Klebsiella pneumoniae*, *Citrobacter youngae*, and *Enterococcus faecalis*.

**Figure 2:**
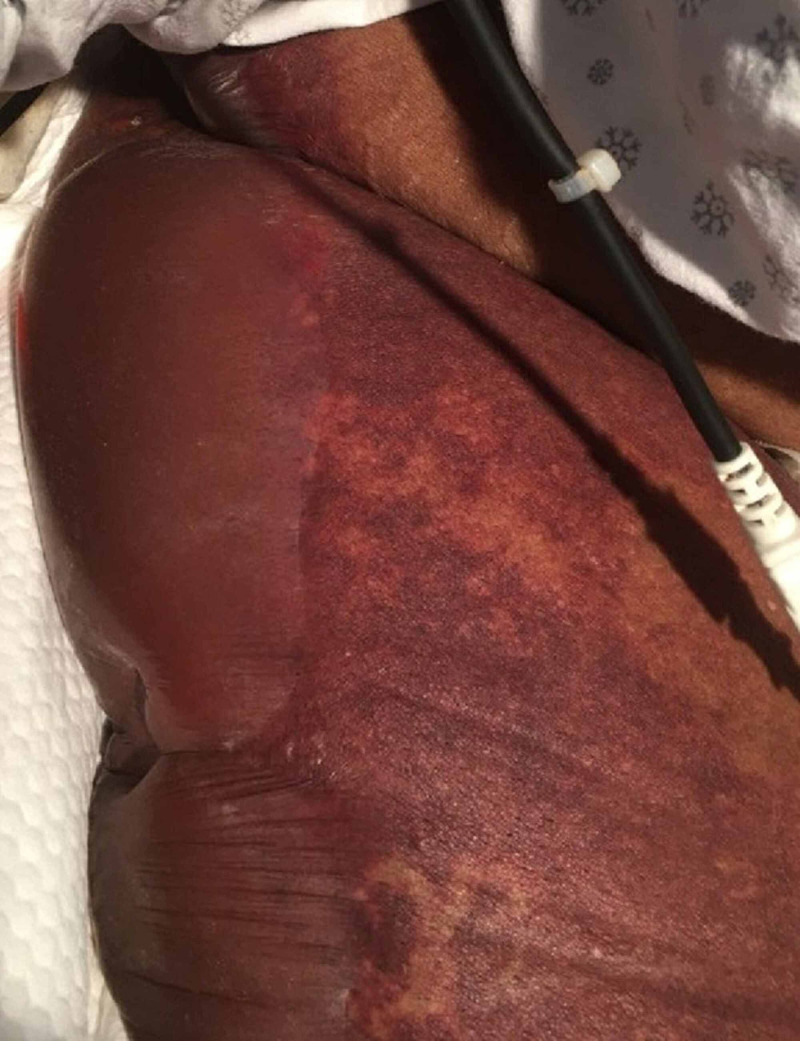
Right thigh on day three.

On day 3, large tense fluid-filled bullae surrounded by retiform purpura were abruptly noted on both legs, all the way up to the pelvic rim (Figure 2). Nikolsky’s sign was present, though there were no mucocutaneous lesions. Labs met the criteria for overt DIC, suggesting acute infectious PF. A coagulation workup revealed only mild decrease in proteins C and S. Therapeutic anticoagulation was started and the patient was transferred to the nearest burn center. There she slowly improved before succumbing to a second episode of PF weeks later.

Two separate instances of histopathology at our institution showed subepidermal blisters with mixed infiltrate and rare necrotic keratinocytes. DIF showed linear staining for IgA at the BMZ. No staining was seen for IgG, IgM, C3, or fibrin. At the burn center, biopsies were done again due to the second episode of PF. Histopathology here showed pauci-inflammatory thrombogenic vasculopathy and necrosis of the epidermis and eccrine coil, with resultant subepidermal bullae and no sign of IgA deposition or any other autoimmune process.

## Discussion

The classic presentation of PF includes erythema, petechiae, and ecchymosis that evolve into painful retiform purpura, usually on the lower extremities and moving proximally. Our patient presented with bullous lesions and denudation with minimal erythema or purpura, making differentiation from TEN challenging.

Clinical progression eventually revealed the classic presentation of PF, but the initial presentation and histopathology complicated the final diagnosis. Linear staining for IgA at the BMZ on DIF suggested the presence of an inflammatory process; however, the third biopsy weeks later showed pauci-inflammatory infiltrate. Given the initial presentation and the stark differences in the disease processes between LABD and PF, we suggest that the patient had an initial instance of spontaneous TEN-like LABD that led to a gram negative sepsis and eventual PF. We feel LABD is the most likely explanation for her sepsis as she lacked any of the common risk factors for gram negative sepsis, such as prior hospitalization, indwelling lines or catheters, or immunosuppression. LABD and its accompanied loss of skin barrier function represents the most likely avenue of infection. While drug-induced LABD is far more likely to cause a TEN-like presentation [6], she was not thought to have been exposed to any new medications in the past year and had lesions preceding her exposure to vancomycin.

## Conclusions

Our report seeks to draw attention to the phenomenon of TEN-like presentations for LABD as well as the severe consequences of such widespread skin barrier dysfunction. This patient was extremely unfortunate to sequentially acquire two rare and ultimately lethal disease processes.
